# Supplementary material for: Exploring the Accuracy and Limits of Algorithms for Localizing Recombination Breakpoints
Source: Mol Biol Evol. 2024 Jun 25;41(7):msae133. doi: 10.1093/molbev/msae133 (PMC11229816; doi:10.1093/molbev/msae133)
Supplement: msae133_Supplementary_Data [file msae133_supplementary_data.zip › supplement.pdf]

Exploring the accuracy and limits of algorithms for localizing recombination breakpoints

Shi Cen[1], David A. Rasmussen[1,2*]

1 Bioinformatics Research Center, North Carolina State University, Raleigh, North Carolina, USA
2 Department of Entomology and Plant Pathology, North Carolina State University, Raleigh, North Carolina, USA

* E-mail: drasmus@ncsu.edu

Supplementary material

1. Site-wise probability derivation

For 3SEQ, the probability of each site being a recombination breakpoint is calculated as the probability of that site reaching the maximum height in a hypergeometric random walk in the single breakpoint case given m and n, the number of informative sites for each parent sequence in sequence triplet. Hence, for each position θ_i, we have

$$P(\theta = \theta_i|m,n) = P(\max \mathbf{H}_{m,n} \leq h_i) = 1 - P(\max \mathbf{H}_{m,n} > h_i) = 1 - \binom{m+n}{n+(h_i+1)} \Big/ \binom{m+n}{n}$$

according to Boni et al. (Barton and Mallows, 1965; Boni *et al.*, 2007).

For MaxChi, the relative site-specific probabilities are derived based on the probability mass function of the hypergeometric distribution and applying the Bayes theorem. Let L be the length of the genome, θ be the inferred position and K be the total number of base pairs with different states. For a specific position $\theta_i \in (0, L)$, let K_{θ_i} be the number of base pairs with different states on the left side of position θ_i. Hence we have the posterior distribution of θ given the sequences and K,

$$\begin{aligned} P(\theta = \theta_i|Sequences, K) &= \frac{Likelihood(Sequences|\theta = \theta_i, K)\pi(\theta)}{m(Sequences)} \\ &\propto Likelihood(Sequences|\theta = \theta_i, K) \\ &= \frac{1}{\binom{\theta_i}{K_{\theta_i}}\binom{L-\theta_i}{K-K_{\theta_i}}} \propto \frac{1}{\frac{\binom{\theta_i}{K_{\theta_i}}\binom{L-\theta_i}{K-K_{\theta_i}}}{\binom{L}{K}}} \\ &= \frac{1}{P(X = K_{\theta_i})}. \end{aligned}$$

where $X \sim \text{Hyper}(K, \theta_i, L)$ and $\theta \sim Unif(0, L)$ based on Smith et al.'s model assumption (Smith, 1992). We used the p-value from the χ^2 test of the 2×2 contingency table to approximate $P(X = K_{\theta_0})$.

For GARD, the site-specific probabilities are given in the program output.

2. Supplementary Figures

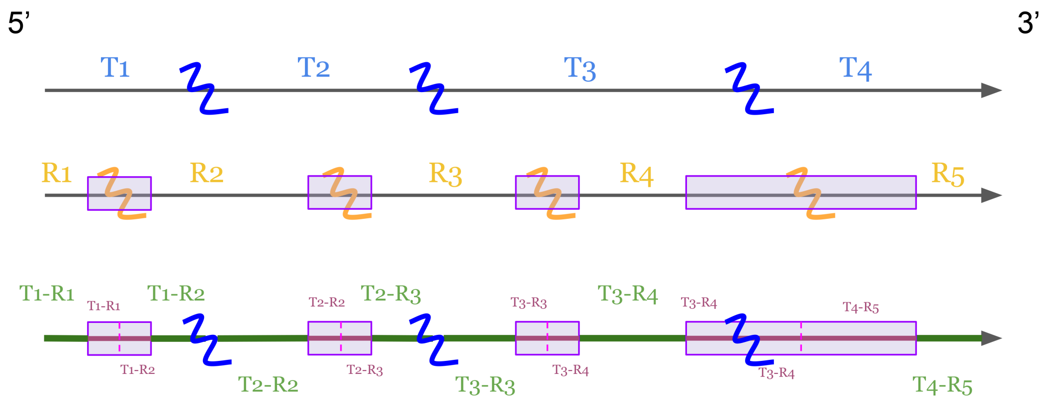

Supplementary Figure 1: Illustration of how Robinson-Foulds distances are computed between true and reconstructed trees. The first alignment is partitioned by true breakpoints, generating three true local trees $(T_1, T_2,$ and $T_3)$. The second alignment is partitioned by inferred breakpoints $(R_1, R_2, R_3$ and $R_4)$. The third alignment shows how RF distance is calculated between true local trees and reconstructed trees.

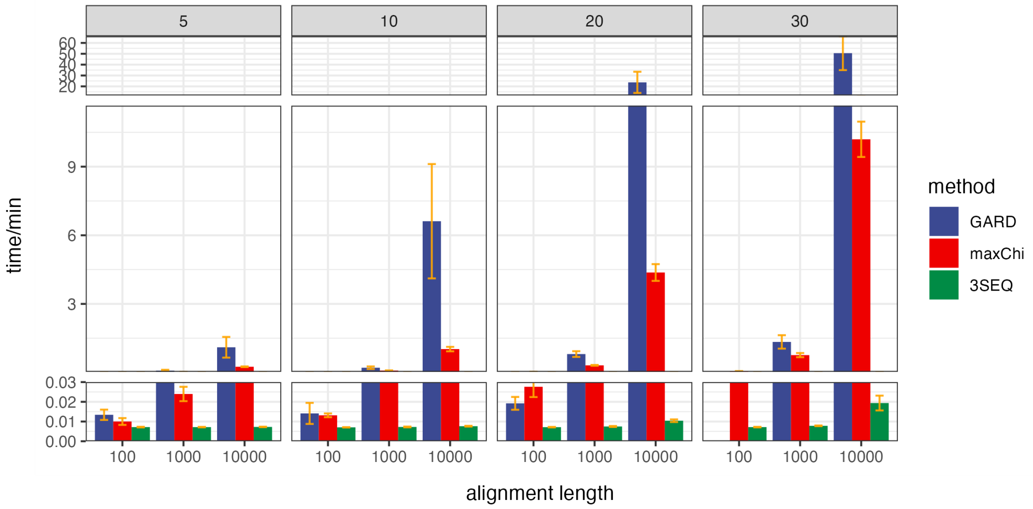

Supplementary Figure 2: Run times for three recombination detection methods on alignments of different sizes (5, 10, 20 and 30 sequences) and lengths (100, 1000 and 10000 bps).

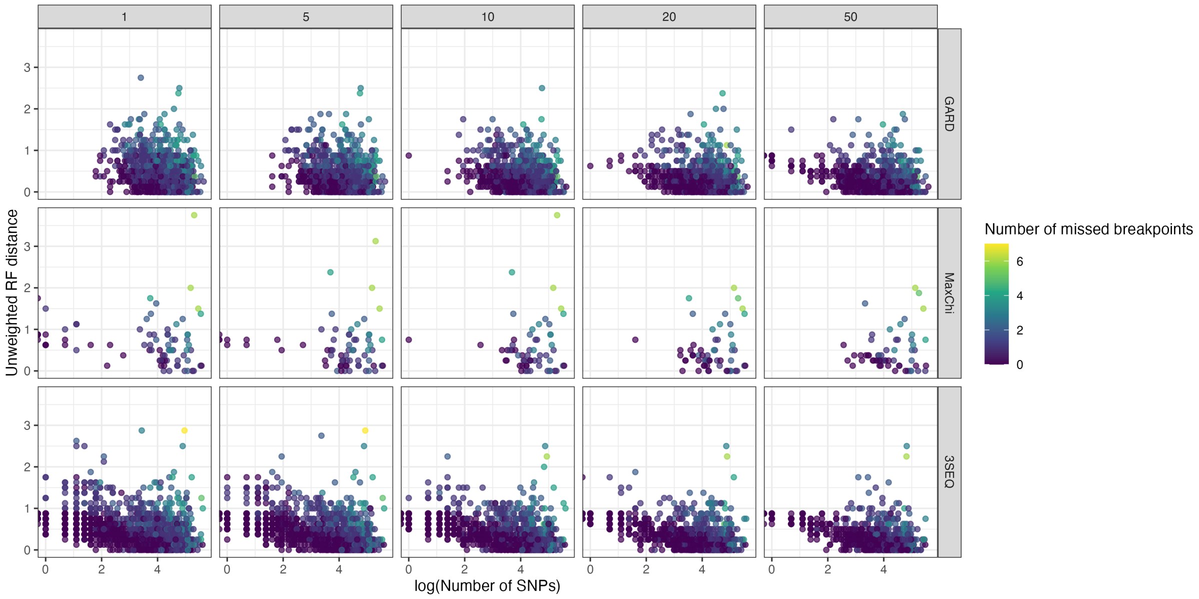

Supplementary Figure 3: The relationship between the number of phylogenetically informative sites (SNPs) and the corresponding unweighted RF distance between the true and reconstructed ML tree for each subalignment. Subalignments were created by partitioning full alignments at breakpoints identified by different detection methods (rows) and using different window sizes (columns) to exclude sites surrounding breakpoints from the resulting subalignments. Points are colored according the the number of true breakpoints that were undetected within each subalignment.

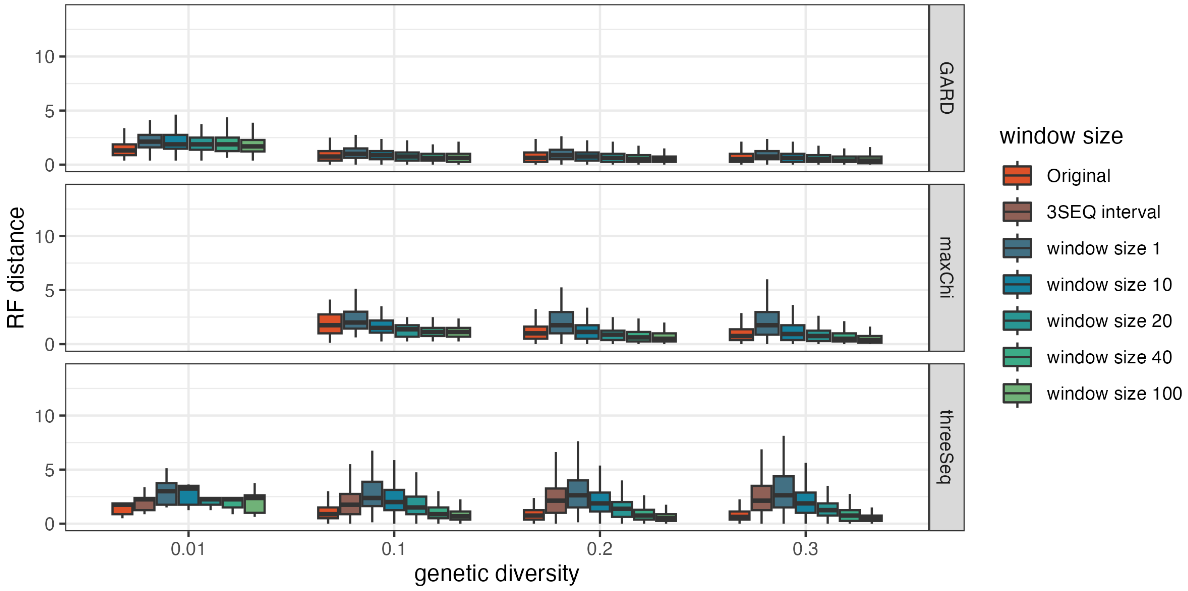

Supplementary Figure 4: Total RF distances between the true and ML trees reconstructed from subalignments split at identified recombination breakpoints using various window sizes. Unlike the results shown in Figure 10 in the main text, the RF distances here are not weighted by the length of the corresponding subalignment, such that the RF distances reflect the accuracy at which individual trees are reconstructed regardless of what proportion of the genome they represent.

References

Barton, D. E. and Mallows, C. L. 1965. Some Aspects of the Random Sequence. *The Annals of Mathematical Statistics*, 36(1): 236 – 260.

Boni, M. F., Posada, D., and Feldman, M. W. 2007. An Exact Nonparametric Method for Inferring Mosaic Structure in Sequence Triplets. *Genetics*, 176(2): 1035–1047.

Smith, J. M. 1992. Analyzing the mosaic structure of genes. *Journal of Molecular Evolution*, 34(2): 126–129.
