# Supplementary figures and images for: Exploring the Accuracy and Limits of Algorithms for Localizing Recombination Breakpoints
Source: Mol Biol Evol. 2024 Jun 25;41(7):msae133. doi: 10.1093/molbev/msae133 (PMC11229816; doi:10.1093/molbev/msae133)

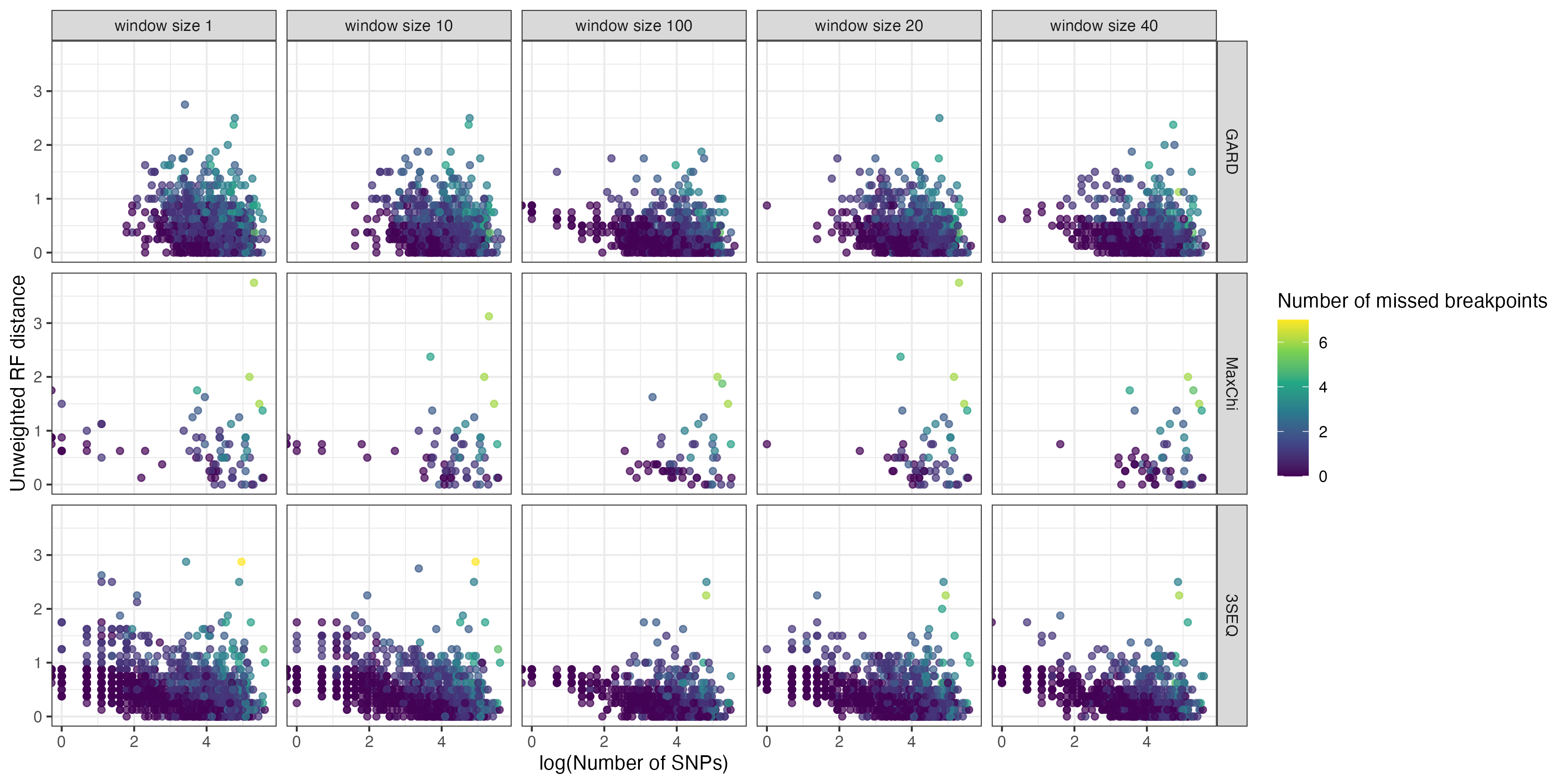

Supplement: msae133_Supplementary_Data [file msae133_supplementary_data.zip › Supplementary_Figure_3.tif]

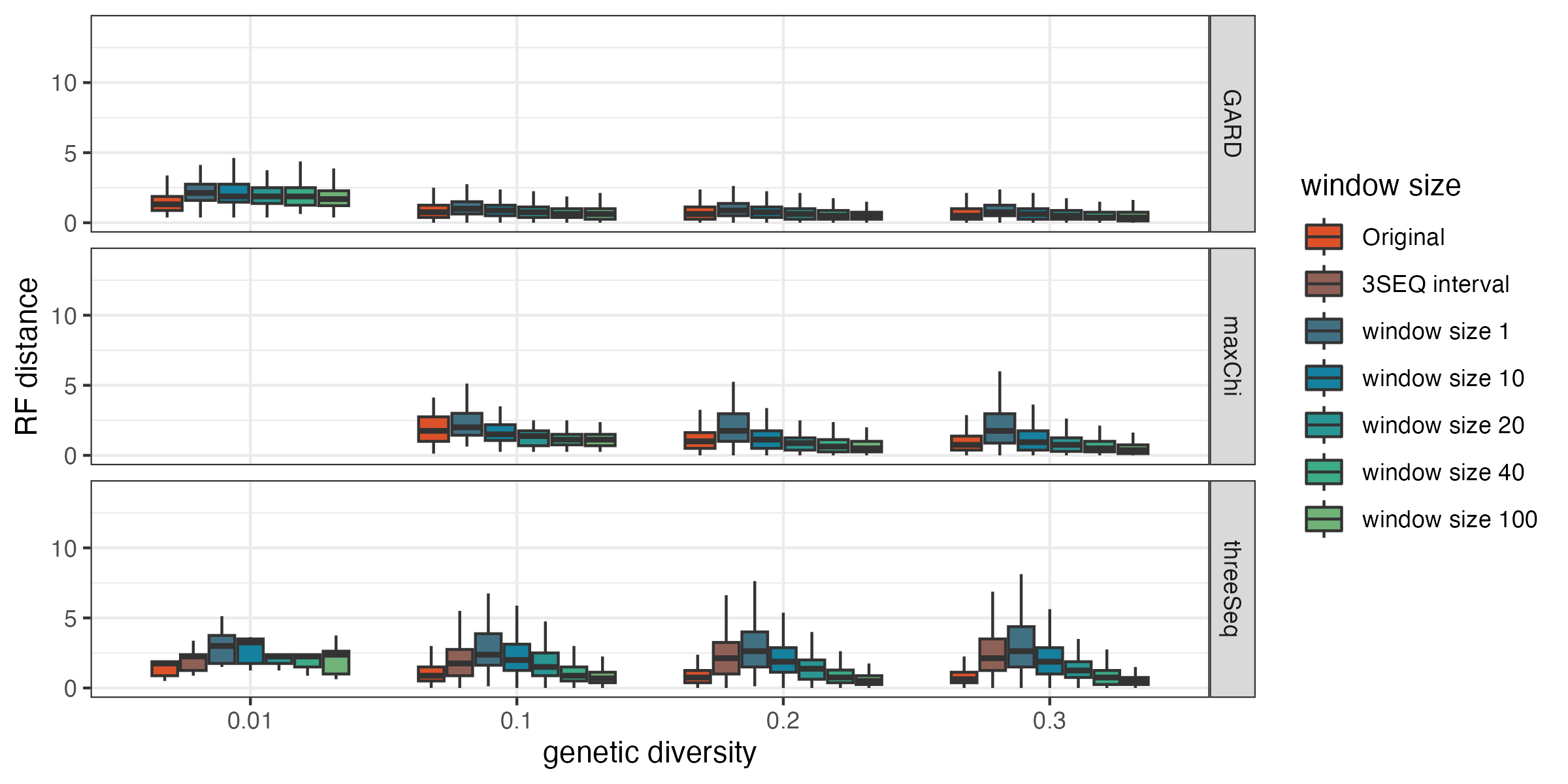

Supplement: msae133_Supplementary_Data [file msae133_supplementary_data.zip › Supplementary_Figure_4.tif]

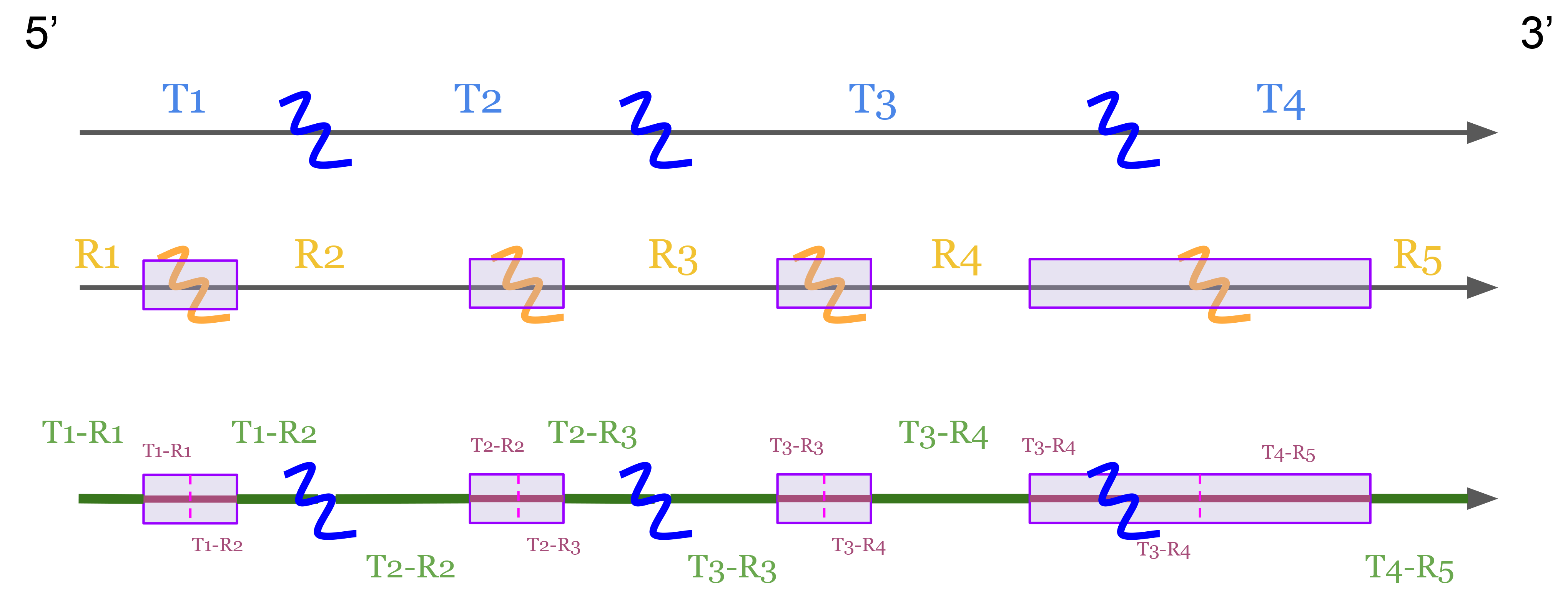

Supplement: msae133_Supplementary_Data [file msae133_supplementary_data.zip › Supplementary_Figure_1.tif]

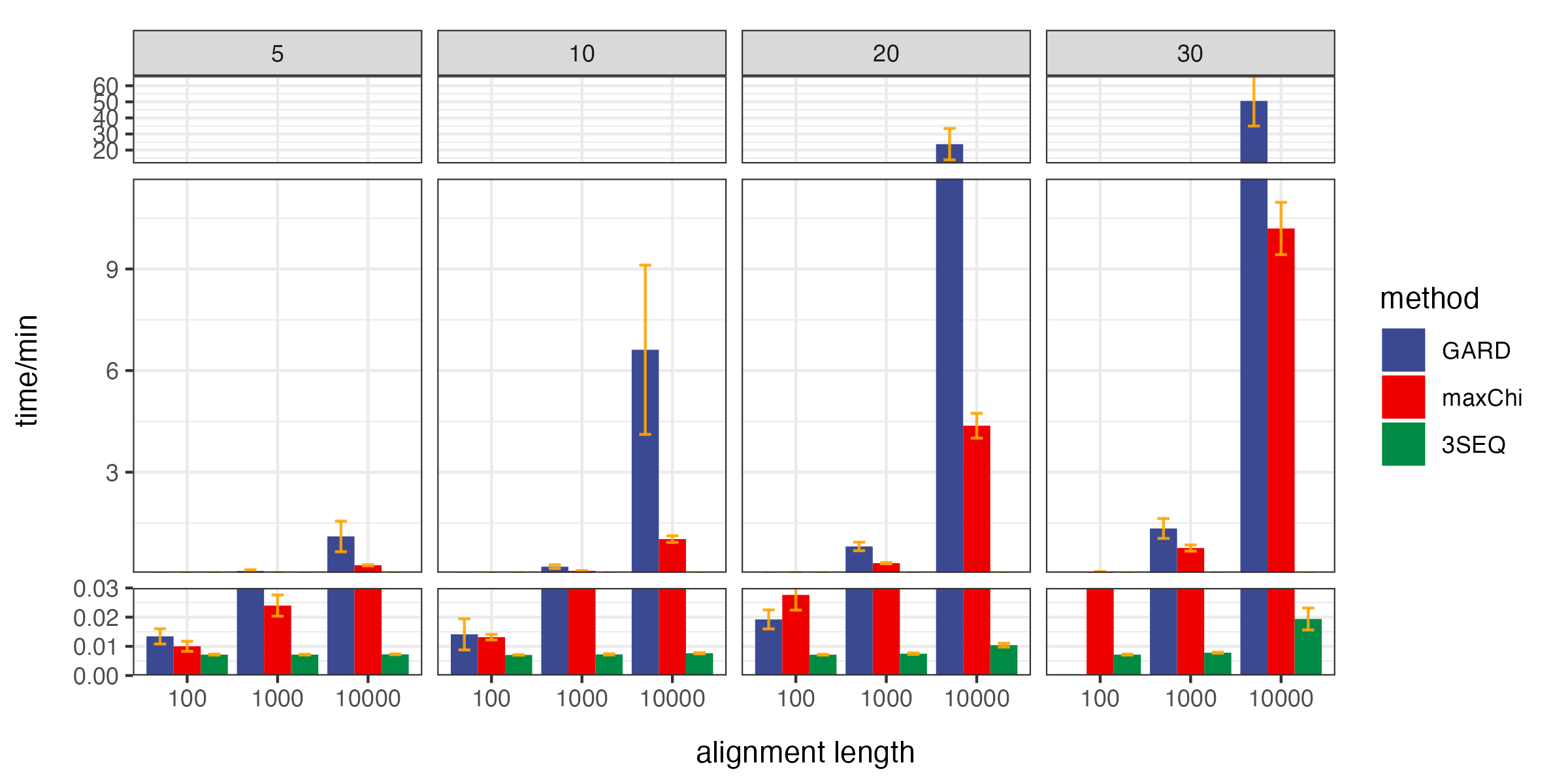

Supplement: msae133_Supplementary_Data [file msae133_supplementary_data.zip › Supplementary_Figure_2.tif]
